# Subcellular distribution of ERK phosphorylation in tyrosine and threonine depends on redox status in murine lung cells

**DOI:** 10.1371/journal.pone.0193022

**Published:** 2018-02-28

**Authors:** Katia E. Helfenberger, Nerina M. Villalba, Bruno Buchholz, Alberto Boveris, Juan José Poderoso, Ricardo J. Gelpi, Cecilia Poderoso

**Affiliations:** 1 Universidad de Buenos Aires, Facultad de Medicina, Departamento de Bioquímica Humana, Buenos Aires, Argentina; 2 CONICET-Universidad de Buenos Aires, Instituto de Investigaciones Biomédicas (INBIOMED), Buenos Aires, Argentina; 3 Universidad de Buenos Aires, Facultad de Medicina, Hospital de Clínicas “José de San Martín”, Laboratorio del Metabolismo del Oxígeno, Buenos Aires, Argentina; 4 CONICET-Universidad de Buenos Aires, Instituto de Inmunología, Genética y Metabolismo (INIGEM), Buenos Aires, Argentina; 5 Universidad de Buenos Aires, Facultad de Medicina, Departamento de Patología, Buenos Aires, Argentina; 6 CONICET-Universidad de Buenos Aires, Instituto de Bioquímica y Medicina Molecular (IBIMOL), Buenos Aires, Argentina; Universidade Federal do Rio de Janeiro, BRAZIL

## Abstract

Activation of ERK1/2 implies the phosphorylation of tyrosine (pTyr) and threonine (pThr) by MEK1/2; both reactions were thought to be cytoplasmic, promoting ERK to reach the nucleus where it activates several transcription factors. In addition, H_2_O_2_ concentrations are known to modulate ERK intracellular translocation, which impacts on cellular proliferation. In this context, the objective of this work was to study the sequence of ERK phosphorylation under two redox conditions and to analyze a putative mitochondrial contribution to this process, in LP07 murine lung cells. A time-course of H_2_O_2_ administration was used and ERK phosphorylation was analyzed in cytosol, mitochondria and nuclei. At 1μM H_2_O_2_, a proliferative redox stimulus, immunoblot revealed a fast and transient increase in cytosol pTyr and a sustained increase in mitochondrial pTyr content. The detection for pThr/pTyrERK (2pERK) showed in cytosol a marked increase at 5 minutes with a fast dephosphorylation after that time, for both H_2_O_2_ concentrations. However, at 50 μM H_2_O_2_, an anti-proliferative condition, 2pERK was gradually retained in mitochondria. Interestingly, these results were confirmed by *in vivo* experiments using mice treated with a highly oxidizing agent [H_2_O_2_]. By the use of two ERK2 mutant constructions, where Tyr and Thr were replaced by alanine, we confirmed that 2pERK relied almost completely on pThr183. Confocal microscopy confirmed ERK subcellular distribution dependence on the incidence of cytosolic pTyr and mitochondrial pThr at 1μM H_2_O_2_. This work shows for the first time, both *in vitro* and *in vivo*, an ERK cycle involving a cross-talk between cytosol and mitochondria phosphorylation events, which may play a significant role in cell cycle progression, proliferation or differentiation under two different redox conditions.

## Introduction

The importance of protein kinases in proliferative or anti-proliferative cellular processes has been extensively reported. In general, the activation of classic members of the mitogen-activated protein kinase (MAPK) family ‒extracellular signal-regulated protein kinases 1/2 (ERK1/2), JNK1/2 and p38MAPK‒ involves single or double phosphorylation reactions, as given by the effects of upstream regulators like MAPK kinases (MAPKKs) called MEK 1–6 [1]. ERKs are produced by two distinct genes; Erk1 (MAPK3) and Erk2 (MAPK1), which are mainly transcribed into proteins p44ERK1 and p42ERK2, but also into several alternatively spliced forms ERK1b, ERK1c, and ERK2a [2]. However, more than a hundred different compounds activate ERK1/2, which in turn participate in different cellular processes like proliferation, differentiation and development, hormone synthesis, stress or learning [3–5]. Of great importance for cellular fate, activated ERKs translocate to the nucleus to phosphorylate multiple transcription factors [6]. This is a well recognized process in ERK signaling cascade [7,8]. Nevertheless, ERKs can also remain in the cytoplasm bound to scaffold proteins, where they phosphorylate a different set of cytoplasmic substrates which also lead to cell proliferation [9]. Furthermore, ERKs can phosphorylate RSK or other substrates which are able to boost ERK-induced phosphorylation events. These activities ultimately lead to the regulation of numerous stimulated cellular processes such as proliferation, differentiation, and survival [2].

MAPKs are unique among the Ser/Thr protein kinases, in the sense that they require both threonine (Thr) and tyrosine (Tyr) phosphorylation for full activation. ERK1/2 activation requires Ras-Raf mediated GTP-dependent double phophorylation in adjacent Tyr and Thr located in the TEY activation motif (Thr-Glu-Tyr) under epidermal growth factor (EGF) stimulus. In particular, the dual phosphorylation of Thr183 and Tyr185 in the ERK2 sequence is catalyzed by MEK1/2 [10] and both events are required for ERK2 to be fully active. The crystal structure of unphosphorylated ERK2 shows Thr183 and Tyr185 to be contained within a loop structure. In this inactivated form, Tyr185 is buried in a hydrophobic pocket and Thr183 is exposed on the molecule surface. As the active form of ERK2 is phosphorylated on both Tyr185 and Thr183, ERK2 must suffer a conformational change in this loop upon association with MEK. Therefore, the amino acid residues contained within this phosphorylation loop are likely to contribute to the conformational subcellular mobility of ERK [11].

Complete inactivation of ERKs can be achieved by the removal of phosphates from either one of the regulatory residues, or from both of them together [12]. Thus, protein Ser/Thr phosphatases, protein Tyr phosphatases, and dual specificity MAPK phosphatases (MKPs) all dephosphorylate ERKs to directly determine the strength and duration of the signals [4,13].

In addition to signaling cascades initiated by hormones or growth factors, reactive oxygen species (ROS) are involved in physiological signaling pathways that regulate a variety of cellular functions. Endogenously generated oxidants such as nitric oxide (NO) and hydrogen peroxide (H_2_O_2_) can act as second messengers, modulating the activities of molecules involved in key cellular processes, including phosphorylative cascades [14]. In particular, H_2_O_2_ is a critical second messenger in fundamental biological processes [15]. An interesting notion is that a continuous increase in oxidant concentration may trigger different cell responses: slight variations in H_2_O_2_ concentration (0.7–20 μM) drive normal cell fate, i.e., proliferation [16,17], arrest, senescence or apoptosis [18].

Increasing evidence shows that MAPKKs are redox-regulated at different levels as well. We have previously reported that high phosphorylated ERK1/2 content is associated with proliferation and low H_2_O_2_ steady-state concentrations ([H_2_O_2_]_ss_) in proliferating embryonic and tumoral tissues, while p38 and JNK1/2 activation is predominantly connected with high [H_2_O_2_]ss, required for tumor arrest [19].

In LP07 murine lung adenocarcinoma cells, cell cycle modulation by H_2_O_2_ is orchestrated by MAPKs, as described by Galli and coworkers [19]. At 1 μM H_2_O_2_, redox-induced cell proliferation is almost totally dependent on ERK activity. Instead, cell cycle arrest (apoptosis excluded) observed with 50 μM H_2_O_2_is specifically mediated by the activation of p38 and JNK1/2. Then, different H_2_O_2_ concentrations are known to promote or inhibit the cellular cycle via these MAPKs [19]. [H_2_O_2_]ss in LP07 murine tumor cells is very low ‒10^−11^ M‒, similarly to what happens in embryonic and proliferating tissues. In these cells, mitochondria have a low H_2_O_2_ production rate but still respond to oxidative stress like normal cell mitochondria do [20,21]. MEK1/2 and ERK1/2 display translocation to mitochondria and traffic to the nucleus in response to treatment with H_2_O_2_ and, ultimately, sustain the phenotype of LP07 tumor cells [19].

In this regard, modulation by H_2_O_2_ entails the entrance of cytosolic phospho-Akt1 Ser473 to mitochondria, where it is further phosphorylated in Thr308 by constitutive kinase PI3K-dependent kinase 1 (PDK1). Moreover, the connection among mitochondrial dysfunction, H_2_O_2_ yield and activation of MAPKs still awaits further elucidation.

In this context, the objective of the present study is to establish the effect of different H_2_O_2_ conditions on the subcellular distribution of the two phosphorylating reactions of ERK structure in Tyr and Thr. For the first time, we demonstrate both *in vitro* and *in vivo* that most of the phosphorylation in Tyr is cytosolic, while phosphorylation in Thr of ERK is mainly mitochondrial, under proliferative H_2_O_2_ conditions. Although ERK2 full activity relies on the addition of phosphoric acid to both Tyr185 and Thr183 in the ERK sequence, we highlight here the relevance of one site or the other by the use of mutants for these two amino acids. Tyr185 is phosphorylated upstream by MEK and, then, Thr183 is more susceptible to phosphorylation in the mitochondrial context, perhaps allowing ERK to further translocate to the nucleus under proliferative conditions. We demonstrate that the redox status modulates the pattern of distribution of phospho-ERK providing a possible mechanistic explanation for cell cycle regulation triggered by oxidant agents such as H_2_O_2_.

## Materials and methods

### Cell line, culture conditions and treatments

The LP07 murine cell line was derived from P07 lung tumor spontaneously developed in a BALB/c mouse and extensively characterized [22,23]. Cells were maintained in Dulbecco’s modified Eagle’s medium nutrient mixture F-12 HAM (D-MEM) from Thermo Fisher Scientific (Waltham, MA, USA) with 10% fetal bovine serum (FBS) and 50 mg/ml gentamycin. For treatments, cells were serum-starved for 24 h and then stimulated with epidermal growth factor (EGF) or H_2_O_2_ (Sigma-Aldrich, St. Louis, MO, USA) for the times indicated in the figures. Sterile and plastic material for tissue culture was from Orange Scientific (Braine-l’Alleud, Belgium). All other reagents were of the highest grade available.

### Isolation of subcellular fractions

Cells were lysed in MSHE buffer (0.22 M mannitol, 0.07 M sucrose, 0.5 mM EGTA, 2 mM HEPES/KOH, 1 mM phenylmethylsulfonylfluoride (PMSF), 5 μg/ml leupeptin, 5 μg/ml pepstatin, 5 μg/ml aprotinin, 25 mM NaF, and 1 mM sodium orthovanadate, pH 7.4) supplemented with a classic inhibitors cocktail (0.22 M mannitol, 0.07 M sucrose, 0.5 mM EGTA, 2 mM HEPES/KOH, 1 mM phenylmethylsulfonylfluoride (PMSF), 5 mg/ml leupeptin, 5 mg/ml pepstatin, 5 mg/ml aprotinin, 25 mM NaF, and 1 mM sodium orthovanadate, pH 7.4). All reagents were purchased from Sigma-Aldrich. The homogenate was centrifuged 10 min at 1000xg (pellet = crude nuclear extract) and 20 min at 10000xg (pellet = mitochondria; supernatant = cytosol). Mitochondria were resuspended in MSHE. The crude nuclear extract was washed with buffer A (10 mM Tris, 1.5 mM EDTA, 10% glycerol, 1mM PMSF, 5 mg/ml leupeptin, 5 mg/ml pepstatin, 5 mg/ml aprotinin, 5 mM NaF, and 1 mM sodium orthovanadate, pH 7.4) containing 0.01% NP-40, resuspended in buffer A plus 0.4 M KCl, and incubated 30 min at 4°C. The suspension was centrifuged 30 min at 105000xg and diluted with buffer A to reduce salt concentration. The purity of the fractions was assessed by western blot with antibodies against translocase outer membrane 40 (TOM40) or complex III (CIII) (mitochondria), actin (cytosol) and Polimerase II or TFIID (TBP) (nuclei). All fractions were tested against crossed contamination with antibodies for other fractions, as previously described [24]. Protein content was determined by Bradford’s method using protein assay reagents from Bio-Rad Laboratories (Hercules, CA, USA).

### Animal care

This study was carried out in strict accordance with the recommendations and the ethical standards of the Animal Care and Research Committee of the University of Buenos Aires (CICUAL, UBA). This protocol was approved by CICUAL-UBA # 0037016/2012. FVB mice were housed in ventilated cages with a 12-hour light/dark cycle and controlled temperature (20–22°C), and fed with normal chow and water *ad libitum*. All surgery was performed under sodium pentobarbital anesthesia, and all efforts were made to minimize suffering.

### Animal treatment and lung tissue samples

Normal male FVB mice (25–35 g) were anesthetized with pentobarbital (90 mg/kg). Once mice were hemodinamically stable, the right yugular vein was dissected and a bolus of 6 mM H_2_O_2_ or saline solution was administered intravenously (*n* = 3 for each treatment). After 5 minutes, mice were euthanized with an overdose of pentobarbital, and the lungs were quickly excised and placed in liquid nitrogen. Lung tissue samples were obtained by mechanical disruption in MSHE buffer using an Ultra-Turrax homogenizer and differential centrifugation: 20 min at 10000xg for the mitochondrial fraction and 1h at 105000xg for the cytosol. Samples were then subjected to western blot studies.

### Western blot

Proteins separated by SDS-PAGE, were transferred onto PVDF membranes and immunoblotted with antibodies anti-double phosphorylated ERK1/2 (dilution 1:5000) (#9101, Cell Signalling Technologies, Danvers, MA, USA) and anti-phosphoTyr ERK (dilution 1:1000) (#sc-7383, Santa Cruz Biotechnology Inc., Dallas, TX, USA). After cell transfection, anti-V5 tag (dilution 1:1000) (#ab 27671, SV5-Pk1, Abcam, Cambridge, UK) was used to detect specifically recombinant over-expressed ERK2 protein. Anti-complex III or TOM40 (dilution 1:5000) (#ab 14745, Abcam, Cambridge, UK) and anti-polimerase II (POLII) or anti-TFIID (or TBP) (#ab sc-421, Santa Cruz Biotechnology Inc., Dallas, TX, USA) (dilution 1:1000) were used as mitochondrial and nuclear loading control respectively, and antibodies against actin (dilution 1:4000) were used for cytosolic fraction characterization (Upstate, Fisher Scientific, Thermo Fischer Scientific, #05661). Cells were then incubated with secondary horseradish peroxidase-conjugated goat anti-rabbit or anti-mouse antibodies (dilution 1:5000; #1706515 and #1721011, respectively; Bio-Rad Laboratories Inc.). Chemiluminescence was developed with enhanced ECL reagent (GE Healthcare, Buckinghamshire, UK) and bands were detected by autoradiography (X100 Autoradiography film, GE Healthcare). Acrylamide and PVDF membranes were from Bio-Rad Laboratories Inc.

### Cell transfection

LP07 cells were seeded onto a 12-well plate and grown to 80% confluency. Twenty-four hours later cells were transiently transfected with 0.4 μg of the plasmids encoding for ERK2 wild-type and ERK2 mutants Y185A or T183A in Opti-MEM Reduced serum medium (Thermo Fisher Scientific) using Lipofectamine 2000 (Life Technologies, Inc., Gaithersburg, MD, USA) according to manufacturer’s instructions (4μl lipofectamine/well). Cells were then placed into normal culture medium 6 h after transfection and grown for further 48 h. After transfection, cells were used as described in the respective figures. Full length ERK2 cDNA was subcloned in the eukaryotic expression plasmid pcDNA3.1 V5-His-TOPO (ThermoFisher Scientific, Waltham, MA, USA) and directed mutagenesis was performed by a biotechnological company (Genscript, Piscataway, NJ, USA).

### Fluorescence labeling and confocal microscopy

Cells were grown on cover slides and transfected as mentioned above. Forty-eight hours post-transfection, cells were incubated with H_2_O_2_ as indicated in the figures, then stained with 100 nM specific mitochondrial marker MitoTracker Deep Red 633 FM (Molecular Probes, Thermo Fisher Scientific) for 45 min at 37°C, fixed in 4% paraformaldehyde, blocked in 1% BSA, 0.3% Triton X-100 PBS, pH 7.4, in a humidified chamber for 1 h, and incubated with primary antibody against V5 tag (anti-V5 tag) and secondary antibody conjugated with Cy3 (dilution 1:400; #111-165-003, Jackson ImmunoResearch Inc., West Grove, PA, USA) for 1h at room temperature in the same buffer. Cover slides were mounted in Fluorescence Mounting Media (Dako, Agilent Technologies, Santa Clara, CA, USA). Confocal laser scanning microscopy was performed with an Olympus FV1000 using a 6361.35 NA oil immersion objective. Excitation filters and emission detected with a PDA device were as follows: GFP, 488 nm excitation, 500–560 nm emission; Cy3, 532 nm excitation, 580±10 nm emission; MitoTracker Deep Red, 633 nm excitation, 650–750 nm emission. Images were obtained with Olympus Fluoview FV10-ASW software and analyzed with DIPimage software (image processing toolbox for Matlab, Delft University of Technology, The Netherlands). MATLAB images (MathWorks, Natick, MA, USA) were analyzed by intensity correlation analysis (ICA) [25,26]. Briefly, if two structures are part of the same complex or are present at the same place, then their staining intensities should vary in synchrony, whereas if they are in different complexes or structures they will exhibit asynchronous staining.

### Statistical analysis

Data are expressed as the mean ± SD and analyzed by one-way analysis of variance (ANOVA), Dunnett’s test and Scheffe’s test. Statistical significance was accepted at *p*< 0.05.

## Results

### ERK2 presence is compartmentalized upon EGF stimulation in LP07 cells

**Fig 1:**
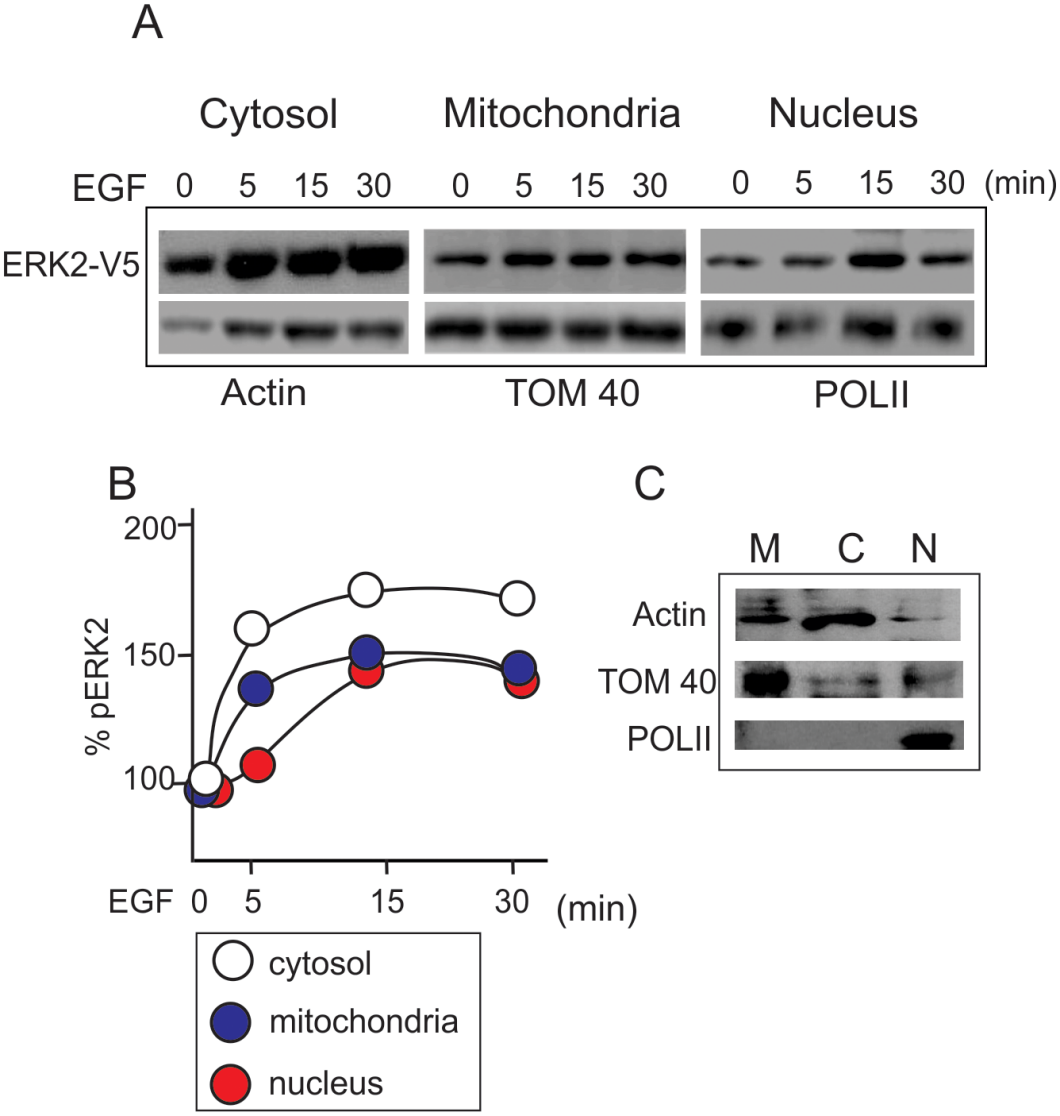
ERK2 is compartmentalized upon EGF stimuli in LP07 tumor lung cells. LP07 cells were transiently transfected with wild type ERK2-V5 (wt), using Lipofectamine 2000 reagent. Twenty-four hours before the experiment, serum was removed from the culture media to avoid any impact on ERK phosphorylation and localization due to serum effect. Forty-eight hours post-transfection, LP07 were stimulated with 10ng/ml EGF peptide for the indicated times. Temporal activation and distribution of ERK2-V5 in the subcellular fractions were analyzed by western blot using antibodies against V5 tag. Representative images of three independent experiments (Panel A). Protein loading was determined with antibodies anti-TOM40 for mitochondria, actin for cytosol, and polymerase II (POLII) for nuclei. In Panel B, the graph indicates amounts of ERK2-V5 respective to control cells (100%). Panel C shows a representative immunoblot with specific antibodies for each subcellular fraction (M = mitochondria; C = cytosol; N = nucleus).

To analyze ERK2 protein kinetics, we incubated LP07 cells for the times indicated with 10ng/ml EGF, which is well known as a classic regulator of the MEK/ERK cascade in several cellular types [27]. Previously, cells were subjected to transient transfection with a plasmid encoding for a recombinant form of ERK2 wild type (wt) fused to a V5 tag (ERK2-V5). The traffic of transfected ERK2-V5 was tracked by western blot with antibodies against V5 tag, with results showing a rapid EGF-promoted increase in ERK2-V5 levels in the cytosol and mitochondria and a slightly delayed increase in the nuclear fraction (Fig 1A). Then, EGF promoted the segregation of ERK2 in specific compartments (Fig 1B) modifying basal ERK2 protein subcellular distribution. This localization pattern is completely in agreement with other extracellular stimuli, like hormone stimulation in steroidogenic cells [5]. Fig 1C depicts the control markers for every fraction, showing little contamination between the subcellular fractions.

### Redox status modulates ERK cellular traffic through differential phosphorylation

Considering that two phosphorylation events are required for ERK full activation, we next analyzed, by means of specific antibodies, the subcellular distribution of phospho-Tyr (pYERK) and the two possible phosphorylations for ERK in Thr or Tyr (2pERK), in LP07 lung tumor cells under two different redox conditions: low (1 μM) and high (50 μM) H_2_O_2_ concentrations.

**Fig 2:**
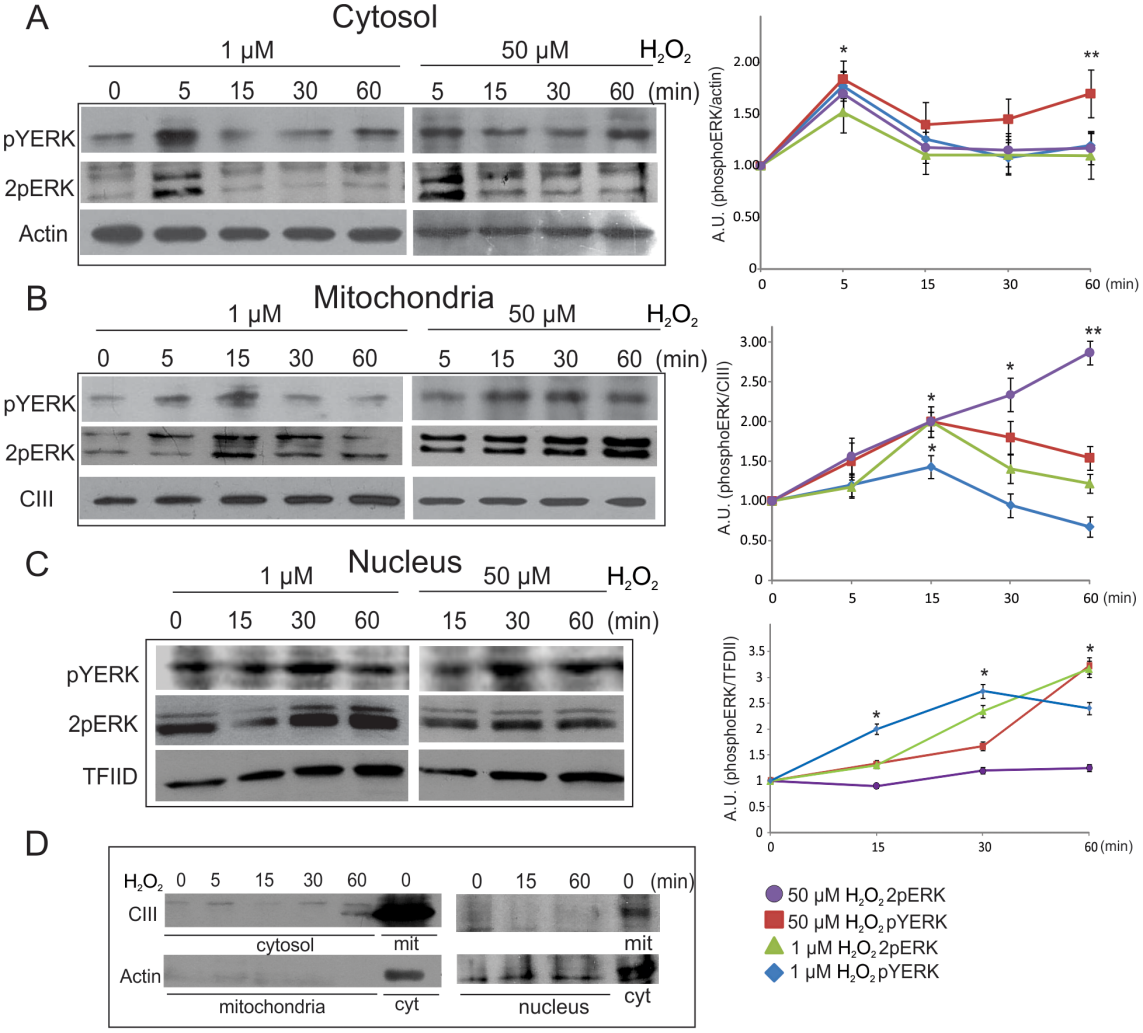
Redox status modulates ERK cellular traffic through differential phosphorylation. LP07 cells were incubated in the presence or absence of 1 μM or 50 μM H_2_O_2_ for the indicated times. Then, mitochondrial, nucleus and cytosolic fractions were obtained and the detection of pTyr (pYERK) and Thr/Tyr phosphorylated ERK1/2 (2pERK) was analyzed by Western blot. Representative images for the cytosolic (Panel A), mitochondrial (Panel B) and nuclear fraction (Panel C) of three independent experiments. Actin, complex III (CIII) and TFIID protein antibodies were used as loading control markers to obtain relative phosphorylated ERK levels. Levels of pYERK and 2pERK relative to actin (Panel A), CIII (Panel B) and TFDII (Panel C) expressed in arbitrary units (A.U.). Panel D shows representative immunoblots with specific antibodies for each subcellular fraction. Data are expressed as the mean ± SD of three independent experiments * p<0.05, **p<0.01 vs. 0 min with H_2_O_2_.

In the presence of 1 μM H_2_O_2_, a rapid and transient peak in pYERK (5 minutes) was detected in the cytosol, with a decrease after 15 min, almost to basal levels (Fig 2A, 1 μM column). This pattern is quite similar for mitochondria, although pTyr peak is detected at 15 minutes (Fig 2B, 1 μM column). When anti-phosphoThr/Tyr antibody was used, an increase was observed in 2pERK levels at 5 minutes of H_2_O_2_ in cytosol and later in mitochondria followed by a fast decrease, especially marked in cytosol (Fig 2A and 2B). When LP07 cells were exposed to 50 μM H_2_O_2_ for different times, it was observed a moderate pYERK peak in the cytosol, but a fast and sustained increase in mitochondria (Fig 2A and 2B, 50 μM column). At this highly oxidative redox status, 2pERK was slowly accumulated in mitochondria and decreased in cytosol after 5 minutes of .H_2_O_2_ (Fig 2A and 2B). Nuclear pYERK and 2pERK signals showed basal phosphorylation of ERK but a modulation after H_2_O_2_ treatment, with a marked and fast increase the presence of the low oxidant concentrations (Fig 2C). Fig 2D depicts a representative immunoblot of cytosol and mitochondria markers on every subcellular fraction. These results suggest that H_2_O_2_ and cellular redox status regulate mitochondrial and cytoplasmic ERK Tyr and Thr phosphorylation which may impact, in turn, on ERK trafficking to the nucleus.

### ERK phosphorylation is mainly regulated in mitochondria under H_2_O_2_ conditions in murine lung tissue

In order to further corroborate the results obtained *in vitro* in LP07 cells, *in vivo* experiments were performed using normal male FVB mice. Animals were treated as described in Materials and Methods and lung tissue samples were used for Western blot studies.

**Fig 3:**
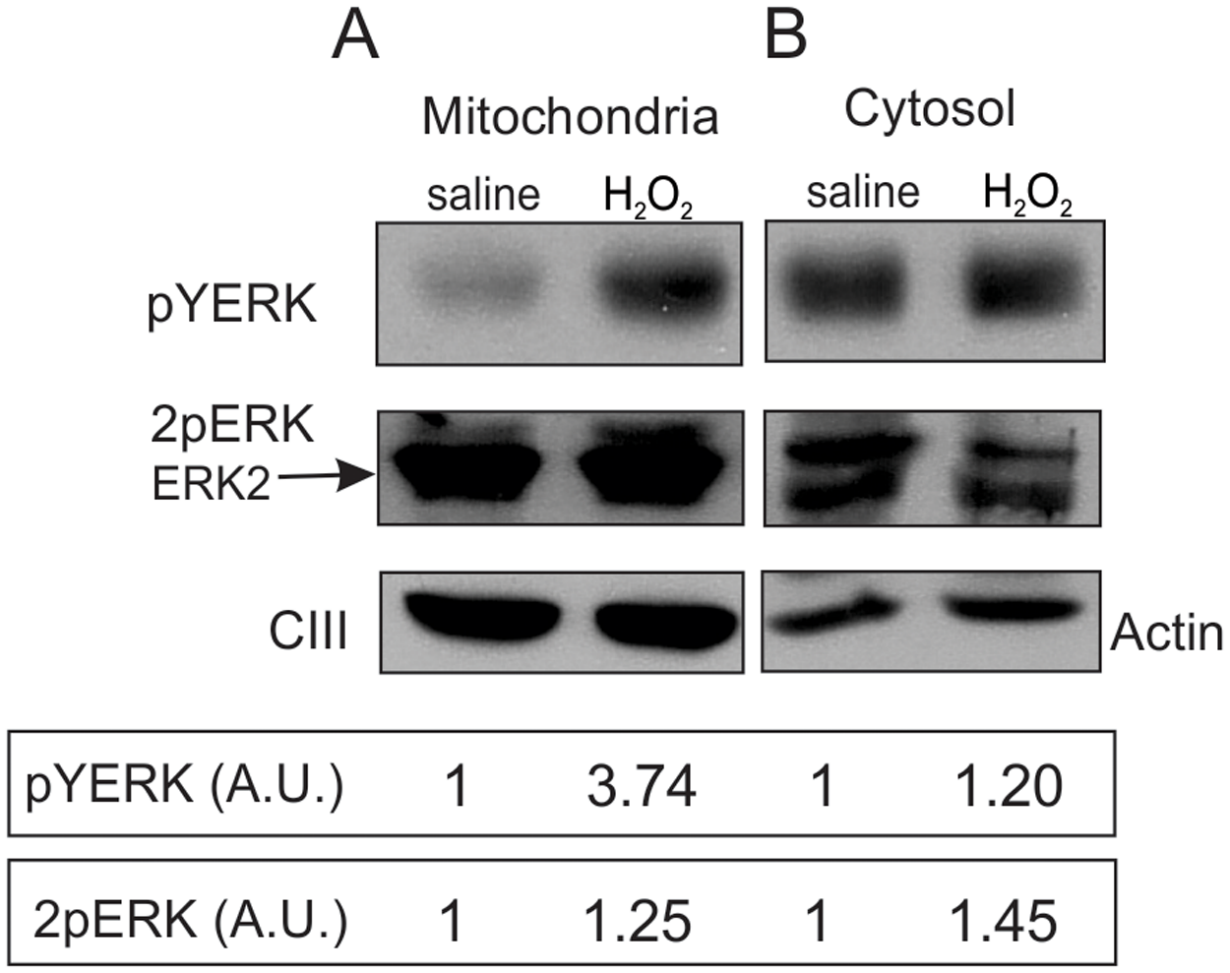
ERK differential phosphorylation depends on H_2_O_2_ in normal murine lung tissue. Normal male FVB mice (*n* = 3) were treated with saline solution or 6 mM H_2_O_2_, both administered intravenously. The detection of pTyr (pYERK) and Thr/Tyr phosphorylated ERK1/2 (2pERK) was analyzed by Western blot. Representative images of the mitochondrial (Panel A) and cytosolic fractions (Panel B) of three independent experiments. Actin and complex III (CIII) antibodies were used as loading control markers to obtain relative phosphorylated ERK levels. Levels of pYERK and 2pERK relative to actin or to CIII expressed in arbitrary units (A.U.). Saline solution-treated mice results were defined as 1 and were used to normalize H_2_O_2_ samples (inset below images).

We detected a marked presence of pTyr (pYERK) in the mitochondrial fraction in mice administered 6 mM of H_2_O_2_ for five minutes, as compared to physiological solution-treated mice (Fig 3A). Instead, the cytosol only showed a slight increase in Tyr phosphorylation after H_2_O_2_ administration (Fig 3B). Some pTyr ERK is detected in cytosol of untreated animals, given that this location is the main compartment of ERK phosphorylation. Nevertheless, the appearance of pTyr ERK2 in mitochondria is entirely dependent on H_2_O_2_ administration.

In addition, high levels of mitochondrial pTyr/pThr (2pERK) were detected in lung tissue from control and H_2_O_2_-treated animals, which suggests 2pERK accumulation in this organelle, while lower 2pERK levels were found in the cytosol, for the same amount of protein analyzed (Fig 3A and 3B). Specifically, a strong band of 2pERK was detected in mitochondria, which matches the molecular weight of ERK2, indicating a strong differential regulation of ERK2 in mitochondria. Then, H_2_O_2_ administration promotes a strong appearance of pTyr signal in mitochondria, suggesting that the translocated pYERK now is double phosphorylated in mitochondria and fully active. Given that ERK2 is the predominant phosphorylated isoform in mitochondria, it can be suggested that ERK2 is the band observed for pTyr in mitochondria (panel A). Results so far show that H_2_O_2_ and redox status also modulate ERK phosphorylation levels and intracellular traffic in normal murine lung tissue.

### Differential Tyr and Thr phosphorylation determines mitochondrial ERK localization

Next, we analyzed the capacity of residues Tyr185 and Thr183 of being phosphorylated and their distribution in mitochondria under H_2_O_2_ challenge. For this purpose, we transiently transfected LP07 tumor cells with ERK2-V5 wt and Y185A and T183A mutant variants, in which Tyr185 and Thr183 were respectively converted to alanine, a non-phosphorylatable amino acid. It is well established that ERK mutants in Tyr phosphorylation site display reduced activity compared to wild type ERK [28].

**Fig 4:**
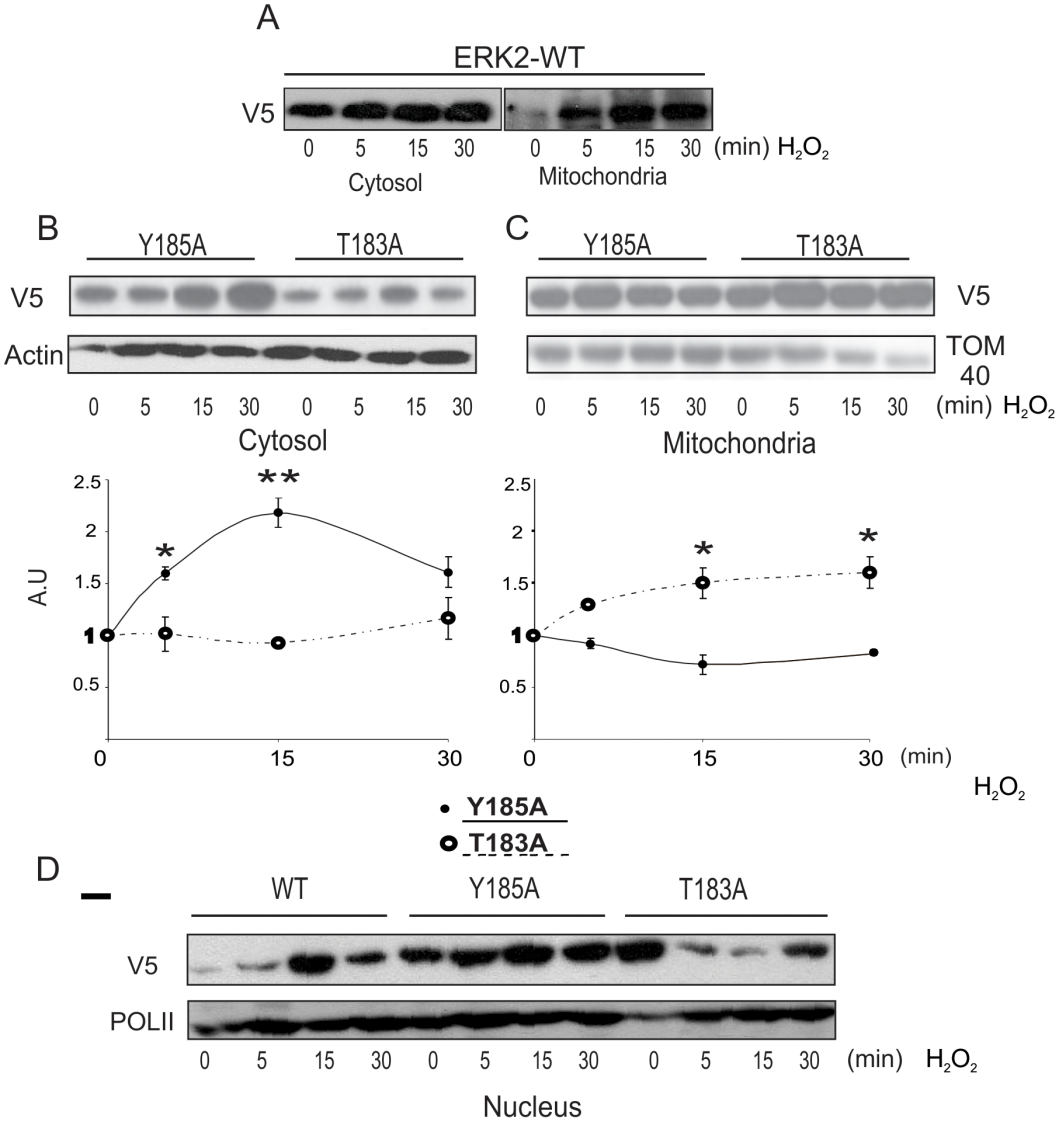
Double phosphorylation of ERK by MEK relies almost completely on mitochondrial phospho-Thr183. LP07 cells were transiently transfected with wild type ERK2-V5 (WT), Y183A or T183A ERK mutants, using Lipofectamine 2000 reagent. Forty-eight hours post-transfection LP07 were stimulated with 1 μM H_2_O_2_ during 0–30 min and then incubated in the presence or absence of 1 μM H_2_O_2_ for the indicated times. Then, the cytosolic, mitochondrial and nuclear fractions were obtained and the detection of ERK2 was analyzed by western blot using anti-V5 antibody. Representative images of cytosolic and mitochondrial fractions ERK2-WT (Panel A) and nuclear fraction ERK2-WT (panel D) distribution and representative images of Y185A and T185A distribution in the cytosolic (Panel B), mitochondrial (Panel C) and nuclear (Panel D) fractions, from three independent experiments. Relative levels of ERK mutants in cytosol and mitochondria relative to basal conditions, arbitrarily defined as 1 (Panels B and C). Data are expressed as the mean ± SD of three independent experiments * p<0.05, **p<0.01 vs. 0 min with H_2_O_2_.

ERK2-V5 wt displayed a quite similar distribution to that obtained for EGF challenge, in agreement with a proliferative cascade triggered by both EGF and H_2_O_2_. This effect was detected in the cytosol, mitochondria and nuclear fraction (Fig 4A). Y185A form accumulated in cytosol and did not significantly increase in the mitochondria of LP07 cells after 1 μM H_2_O_2_ incubation for different times (Fig 4B). In contrast, V5 signal did not increase in the cytosol when samples were obtained from T183A transfected cells, but showed time-dependent accumulation in mitochondria (Fig 4B). Y185A was still found in the nuclear fraction but the presence of T183A provoked a marked decrease in ERK2 access to the nucleus (Fig 4C).

**Fig 5:**
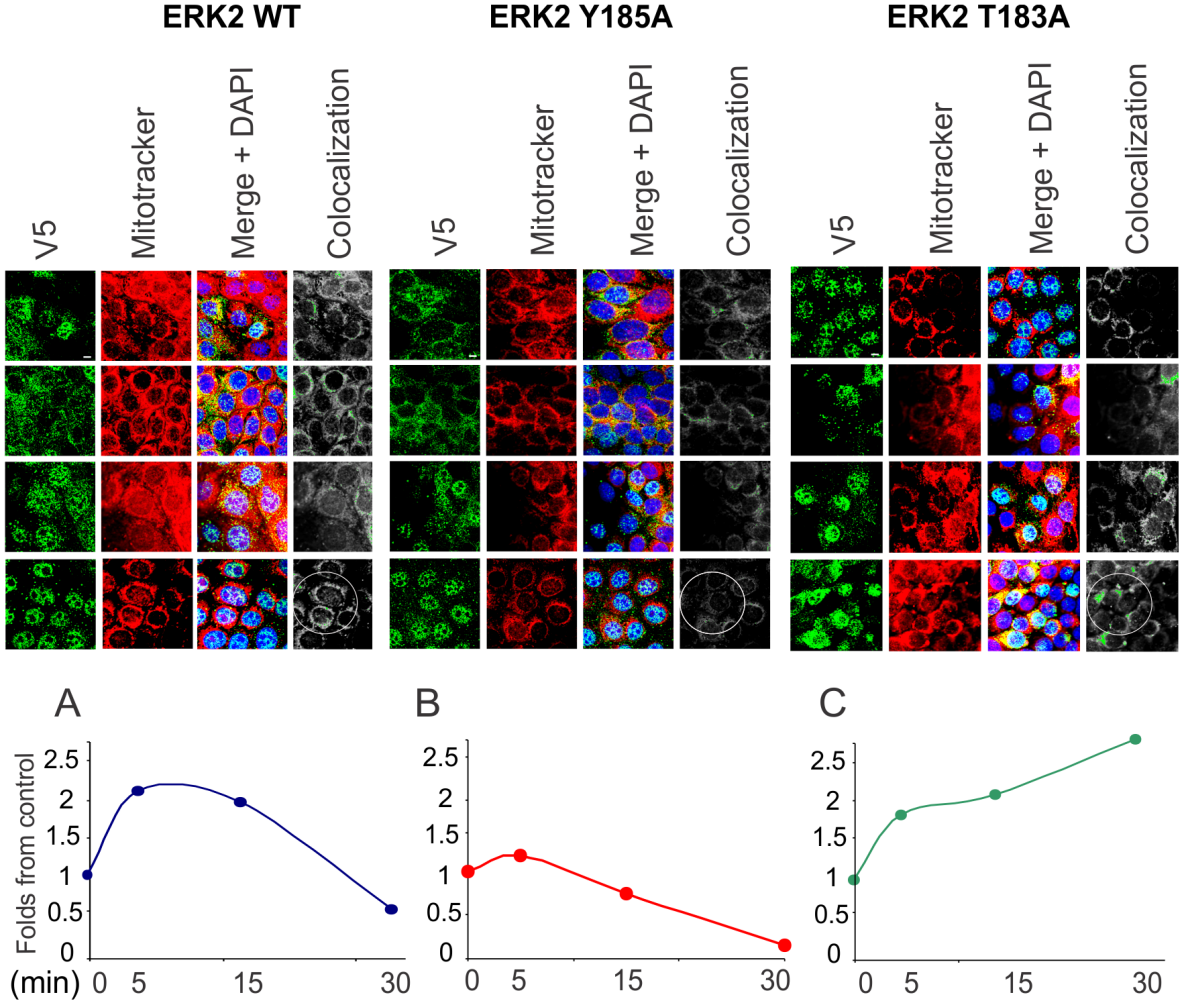
Mitochondrial ERK localization depends on differential phosphorylation. Representative confocal images of LP07 tumor lung transfected cells with wild type ERK/V5 WT (left panel), Y183A/V5 (middle panel) and T183A/V5 (right panel). Forty-eight hours post transfection, cells were incubated with 1 μM H_2_O_2_ during 0–30 min. From top to bottom of the panels images show increasing H_2_O_2_ incubating times. Mitochondria were visualized in red by staining with Mitotracker Deep Red. Cells were fixed and incubated with anti-V5 antibody and a secondary antibody conjugated with Cy2, and analyzed in an Olympus FV1000 confocal microscope. Co-localization was highlighted in green on the grayscale images (last column of each panel). Images directly exported from Olympus Fluoview acquisition program. Fold changes relative to control images of combined MitoTracker and Cy2 intensity for ERK2 WT (Panel A), Y185A (Panel B) and T183A (Panel C). Basal conditions without H_2_O_2_ arbitrarily defined as 1. Bar = 10 μm.

For further confirmation, the behavior of these ERK forms was analyzed by confocal microscopy in a time-course interval ranging from 5 to 30 min of 1 μM H_2_O_2_ incubation. ERK2-V5 wt cells showed a marked V5 and mitochondria signal juxtaposition at short times of H_2_O_2_ incubation, with a peak of more than one-fold ERK activation in the first 15 min (Fig 5A). In contrast, this overlap was rather poor in the Y185A mutant, indicating Tyr185 phosphorylation is indeed crucial to maintain ERK2 mitochondrial activity (Fig 5B). However, T183A mutants restored high levels of mitochondrial ERK2, which suggests that the absence of Thr183 phosphorylation determines that not completely phosphorylated ERK2 accumulates progressively in mitochondria (Fig 5C).

Together, these results indicate that phosphorylation of Tyr is a key event for ERK entering mitochondria to achieve complete activation by mitochondrial MEK phosphorylating ERK in Thr and then translocate to the nucleus. A partially phosphorylated ERK could be more susceptible to mitochondrial phosphatases and remain accumulated without activity.

## Discussion

In the present study we provide novel evidence supporting H_2_O_2_ modulation of specific ERK subcellular distribution and activation in LP07 lung tumor cells. Furthermore, the mechanism operating in ERK activity regulation is also present in murine lung tissue, highlighting the fact that redox conditions are completely relevant for the development of normal cells. Opposite effects elicited by low and high H_2_O_2_ concentrations have been previously observed in various tumor cell lines [29], as well as in normal tissues [30]; these findings reinforce the notion that an increase in H_2_O_2_ concentration can change cell phenotype by eliciting sequential and contrary responses. In LP07 cells, these processes rely on the alternative activation of either ERK1/2 or JNK1/2 and p38 MAPKs by the modulation of the interaction with their cognate MAPKKs, and on the redistribution of these kinases between compartments [19].

Mitochondria are also suitable integration platforms for these signaling pathways due to their key role in cellular metabolism, redox balance, and survival-death events. A large body of evidence indicates that, among other kinases like PKA, PKC and Akt [3,5,24,31,32], MAPKs are present in mammal cell mitochondria, although the precise mechanism by which they are translocated and regulated in the mitochondria remains to be elucidates, as neither MEK1/2 nor ERK1/2 contain mitochondrial leader peptides.

The findings presented here indicate that mitochondrial uptake of ERK phosphorylated in Tyr is required for sequential and immediate phosphorylation in Thr to become fully activated and released to nucleus, in redox-permissive low H_2_O_2_ conditions. In this particular condition, the thiol groups of ERK2 cysteines (Cys382 and Cys214) are oxidized to sulfinic (–SO_2_H) and sulfonic acid (–SO_3_H), as previously described [19]. Mutation of Cys214 to negatively charged glutamic acid enhances MEK-ERK interaction, which indicates that negative charges are involved in this interaction and may imply that cysteine oxidation introduces negative changes in ERK. Afterwards, ERK is distributed across the cytosol, mitochondria and nucleus displaying a redox-dependent activation cycle. Then, under low H_2_O_2_, a strong MEK-ERK association is favored and sequential phosphorylation in both Tyr and Thr residues may contribute to further addition of negative charges and to the aforementioned ERK activation cycle. On the other hand, mutation of Cys214 to alanine rendered ERK2 and MEK accumulation in mitochondria and a decrease in the mitochondrial cycle, in agreement with a lesser extent of negative charge in ERK molecule [19]. This is consistent with the fact that phospho-ERK is accumulated in mitochondria and its nuclear translocation is prevented when Thr phosphorylation is impaired (Fig 5). This effect may reflect ERK modulation at high H_2_O_2_ and provide an explanation for ERK retention in mitochondria, where it could be dephosphorylated and inactivated by mitochondrial phosphatases [4].

These results are completely in line with a classic study by Dr. Cobb’s group, which elegantly demonstrates that the accumulation of pTyr precedes the accumulation of pThr in *in vitro* ERK activity assays [33]. Here, we provide for the first time a mechanistic explanation for these previous and relevant findings on ERK regulation.

MAPK signaling pathway has been extensively explored and transient and sustained ERK activation seems to be one of the most relevant events to direct cellular fate, e.g. transient activation of ERK in the cytoplasm of PC12 cells is essential for proliferation but sustained ERK activation and nuclear accumulation are both required for PC12 cell differentiation [34].

Previous work [5,19,35,36] and this work itself show that not only ERK temporal activation but also ERK compartmentalization modulated by free radicals could determine cellular fate. Particularly in tumor cells, mitochondrial ERK activation is sustained by selective MAP oxidation and desensitizes the permeability transition pore [30], which constitutes a possible mechanistic basis for the contribution to a tumor phenotype and increased resistance to apoptosis of neoplasic cells.

Findings regarding mitochondrial redox influence on ERK activation shown in Fig 2 indicate that, depending on the different ERK phosphorylated forms, mitochondria have a transient participation in ERK activation; the organelles contribute to the redox modulation of the cell cycle and the increase in redox status leads to monophosphorylated ERK accumulation in mitochondria. This novel mechanism is also observed in normal lung murine tissue.

**Fig 6:**
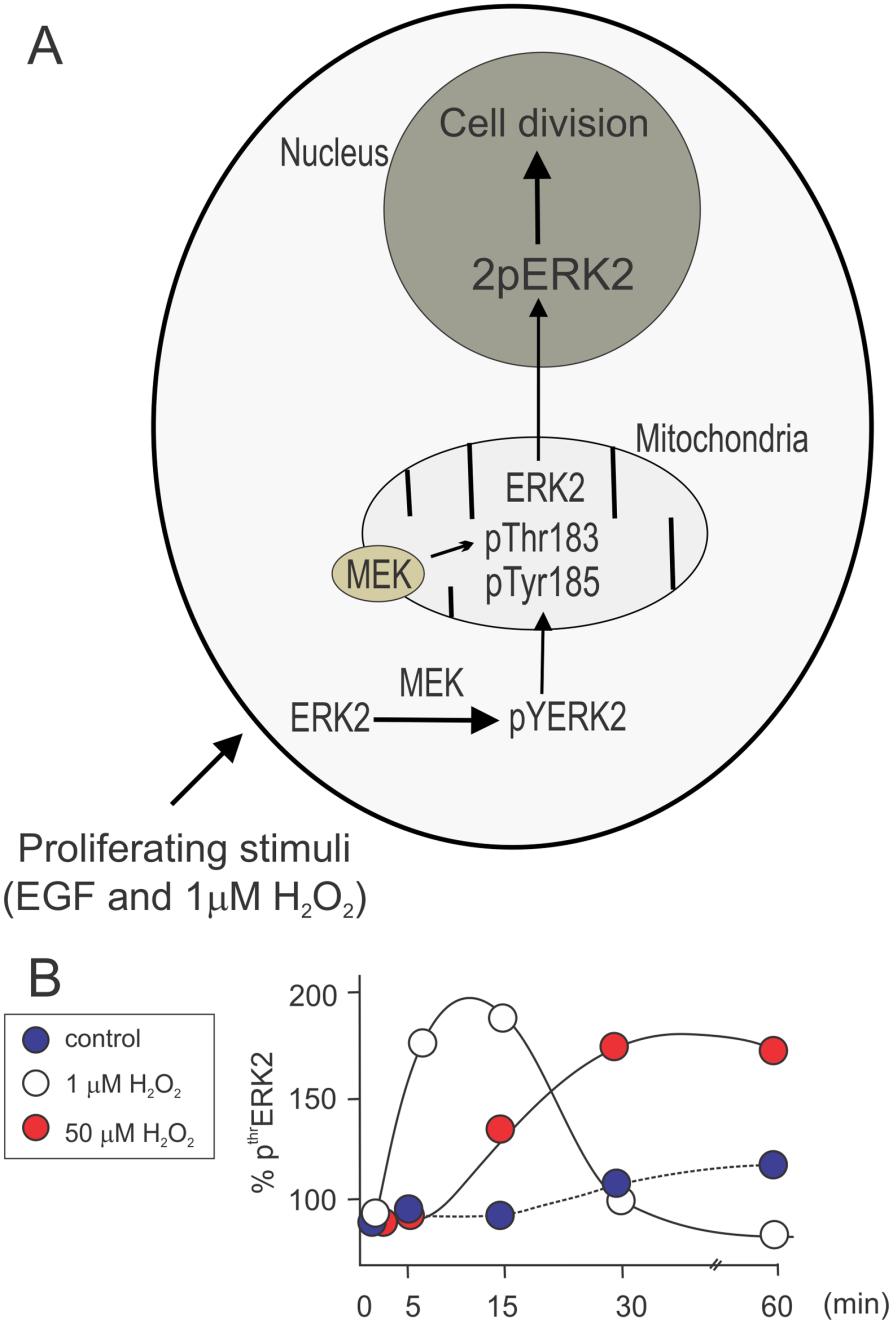
Summary of the proposed mechanistic design for double ERK phosphorylation in cytosol and mitochondria under different redox conditions. After EGF or 1 μM H_2_O_2_ stimulation, which exert a well described and robust proliferative action, ERK2 is rapidly phosphorylated by cytosolic MEK in Tyr185 (pYERK2), allowing a further association with mitochondria and phosphorylation of Thr183, by a mitochondrial MEK pool. This second and compartmentalized phosphorylation drives fully active-ERK2 (2pERK2) translocation to the nucleus to promote gene transcription and cellular division (A). Panel B shows mitochondrial pThr ERK2 kinetics in response to 1 and 50 μM H_2_O_2_. At 1 μM H_2_O_2_ Thr183 is rapidly phosphorylated in mitochondria with a transient localization and fast translocation to the nucleus (white circles); on the other hand, in the presence of 50 μM H_2_O_2_ arresting cell cycle, Thr183 phosphorylated signal is retained in the mitochondrial milieu and ERK translocation to the nucleus is impeded.

Fig 6 shows a schematic diagram where proliferative stimuli such as EGF or 1 μM H_2_O_2_ promotes ERK phosphorylation involving three subcellular compartments, i.e. cytosol, mitochondria and nucleus. A temporal Thr phosphorylation pattern indicates dependence on cellular redox status for the occurrence of this phosphorylation and mitochondrial contribution. It is highlighted here that two amino-acidic residues in close proximity such as Tyr and Thr in the ERK sequence can be modified in a compartmentalized manner, with direct implications for cellular fate and, in turn, cell survival or death. Given the well-established relationship between mitochondria and cancer, these studies appear to provide clues to unveil the machinery that integrates the signals for the acquisition of a malignant phenotype.

## References

[1] Pearson G, Robinson F, Beers Gibson T, Xu BE, Karandikar M, Berman K, et al Mitogen-activated protein (MAP) kinase pathways: regulation and physiological functions. Endocr Rev [Internet]. 2001;22(January):153–83. Disponible en: http://www.ncbi.nlm.nih.gov/pubmed/11294822 10.1210/edrv.22.2.0428 11294822

[2] Bendetz-Nezer S, Seger R. Role of non-phosphorylated activation loop residues in determining ERK2 dephosphorylation, activity, and subcellular localization. J Biol Chem. 2007;282(34):25114–22. doi: 10.1074/jbc.M703120200 17597065 10.1074/jbc.M703120200

[3] Alonso M, Melani M, Converso D, Jaitovich A, Paz C, Carreras MC, et al Mitochondrial extracellular signal-regulated kinases 1/2 (ERK1/2) are modulated during brain development. J Neurochem [Internet]. 2004;89(1):248–56. Disponible en: http://www.ncbi.nlm.nih.gov/pubmed/15030409 10.1111/j.1471-4159.2004.02323.x 15030409

[4] Paz C, Cornejo Maciel F, Gorostizaga A, Castillo AF, Mori Sequeiros García MM, Maloberti PM, et al Role of Protein Phosphorylation and Tyrosine Phosphatases in the Adrenal Regulation of Steroid Synthesis and Mitochondrial Function. Front Endocrinol (Lausanne) [Internet]. 2016;7(June):1–15. Disponible en: http://journal.frontiersin.org/Article/10.3389/fendo.2016.00060/abstract 10.3389/fendo.2016.00060 PMC4899475 27375556

[5] Poderoso C, Converso DP, Maloberti P, Duarte A, Neuman I, Galli S, et al A mitochondrial kinase complex is essential to mediate an ERK1/2-dependent phosphorylation of a key regulatory protein in steroid biosynthesis. PLoS One. 2008;3(1). 10.1371/journal.pone.0001443 PMC2175533 18197253

[6] Lenormand P, Pages G, Sardet C, L´Allemain G, Meloche S, Pouysségur J. MAP kinases: activation, subcellular localization and role in the control of cell proliferation. Adv Second Messenger Phosphoprot Res. 1993;28:237–44. 8398409

[7] Brunet A, Roux D, Lenormand P, Dowd S, Keyse S, Pouysségur J. Nuclear translocation of p42/p44 mitogen-activated protein kinase is required for growth factor-induced gene expression and cell cycle entry. EMBO J [Internet]. 1999;18(3):664–74. Disponible en: http://www.ncbi.nlm.nih.gov/pubmed/9927426%5Cnhttp://www.pubmedcentral.nih.gov/articlerender.fcgi?artid=PMC1171159 10.1093/emboj/18.3.664 PMC1171159 9927426

[8] Buscà R, Pouysségur J, Lenormand P. ERK1 and ERK2 Map Kinases: Specific Roles or Functional Redundancy? Front Cell Dev Biol [Internet]. 2016;4(June):53 Disponible en: http://journal.frontiersin.org/Article/10.3389/fcell.2016.00053/abstract 10.3389/fcell.2016.00053 PMC4897767 27376062

[9] Casar B, Pinto A, Crespo P. Essential Role of ERK Dimers in the Activation of Cytoplasmic but Not Nuclear Substrates by ERK-Scaffold Complexes. Mol Cell. 2008;31(5):708–21. doi: 10.1016/j.molcel.2008.07.024 18775330 10.1016/j.molcel.2008.07.024

[10] Zhou B, Wang Z-X, Zhao Y, Brautigan DL, Zhang Z-Y. The specificity of extracellular signal-regulated kinase 2 dephosphorylation by protein phosphatases. J Biol Chem [Internet]. 2002;277(35):31818–25. Disponible en: http://www.ncbi.nlm.nih.gov/pubmed/12082107 10.1074/jbc.M203969200 12082107

[11] Butch ER, Guan KL. Characterization of ERK1 activation site mutants and the effect on recognition by MEK1 and MEK2. J Biol Chem. 1996;271(8):4230–5. 8626767 10.1074/jbc.271.8.4230

[12] Goldsmith EJ, Akella R, Min X, Zhou T, Humphreys J. Substrate and docking interactions in serine/threonine protein kinases. Chem Rev. 2007;107:5065–81. doi: 10.1021/cr068221w 17949044 10.1021/cr068221w PMC4012561

[13] Kidger AM, Keyse SM. The regulation of oncogenic Ras/ERK signalling by dual-specificity mitogen activated protein kinase phosphatases (MKPs). Vol. 50, Seminars in Cell and Developmental Biology. 2016 p. 125–32. 10.1016/j.semcdb.2016.01.009 PMC5056954 26791049

[14] Bhat NR, Zhang P. Hydrogen peroxide activation of multiple mitogen-activated protein kinases in an oligodendrocyte cell line: role of extracellular signal-regulated kinase in hydrogen peroxide-induced cell death. J Neurochem. 1999;72:112–9. 9886061 10.1046/j.1471-4159.1999.0720112.x

[15] Woolley JF, Corcoran A, Groeger G, Landry WD, Cotter TG. Redox-regulated growth factor survival signaling. Antioxid Redox Signal [Internet]. 2013;19(15):1815–27. Disponible en: http://online.liebertpub.com/doi/abs/10.1089/ars.2012.5028 10.1089/ars.2012.5028 23198948

[16] Davies K. The Broad Spectrum of Responses to Oxidants in Proliferating Cells: A New Paradigm for Oxidative Stress. IUBMB Life [Internet]. 1999;48(1):41–7. Disponible en: http://doi.wiley.com/10.1080/713803463 10.1080/713803463 10791914

[17] Ramsey MR, Sharpless NE. ROS as a tumour suppressor? Nat Cell Biol. 2006;8(11):1213–5. doi: 10.1038/ncb1106-1213 17077852 10.1038/ncb1106-1213

[18] Antunes F, Cadenas E. Cellular titration of apoptosis with steady state concentrations of H2O2: Submicromolar levels of H2O2 induce apoptosis through fenton chemistry independent of the cellular thiol state. Free Radic Biol Med. 2001;30(9):1008–18. 11316581 10.1016/s0891-5849(01)00493-2

[19] Galli S, Arciuch VGA, Poderoso C, Converso DP, Zhou Q, de Kier Joffé EB, et al Tumor cell phenotype is sustained by selective MAPK oxidation in mitochondria. PLoS One. 2008;3(6). 10.1371/journal.pone.0002379 PMC2398776 18545666

[20] Boveris A, Costa LE, Poderoso JJ, Carreras MC, Cadenas E. Regulation of mitochondrial respiration by oxygen and nitric oxide. Ann N Y Acad Sci. 2000;899:121–35. 10863534 10.1111/j.1749-6632.2000.tb06181.x

[21] Carreras MC, Franco MC, Peralta JG, Poderoso JJ. Nitric oxide, complex I, and the modulation of mitochondrial reactive species in biology and disease. Vol. 25, Molecular Aspects of Medicine. 2004 p. 125–39. 10.1016/j.mam.2004.02.014 15051322

[22] Galli S, Labato MI, Bal de Kier Joffé E, Carreras MC, Poderoso JJ. Decreased mitochondrial nitric oxide synthase activity and hydrogen peroxide relate persistent tumoral proliferation to embryonic behavior. Cancer Res. 2003;63(19):6370–7. 14559826

[23] Urtreger AJ, Diament MJ, Ranuncolo SM, Del C Vidal M, Puricelli LI, Klein SM, et al New murine cell line derived from a spontaneous lung tumor induces paraneoplastic syndromes. Int J Oncol. 2001;18(3):639–47. 11179499 10.3892/ijo.18.3.639

[24] Arciuch VGA, Galli S, Franco MC, Lam PY, Cadenas E, Carreras MC, et al Akt1 intramitochondrial cycling is a crucial step in the redox modulation of cell cycle progression. PLoS One. 2009;4(10). 10.1371/journal.pone.0007523 PMC2761088 19844585

[25] Nicoletti I, Migliorati G, Pagliacci MC, Grignani F, Riccardi C. A rapid and simple method for measuring thymocyte apoptosis by propidium iodide staining and flow cytometry. J Immunol Methods. 1991;139(2):271–9. 1710634 10.1016/0022-1759(91)90198-o

[26] Kreft M, Milisav I, Potokar M, Zorec R. Automated high through-put colocalization analysis of multichannel confocal images. Comput Methods Programs Biomed. 2004;74(1):63–7. doi: 10.1016/S0169-2607(03)00071-3 14992827 10.1016/S0169-2607(03)00071-3

[27] Pierce KL, Luttrell LM, Lefkowitz RJ. New mechanisms in heptahelical receptor signaling to mitogen activated protein kinase cascades. Oncogene [Internet]. 2001;20:1532–9. Disponible en: http://www.ncbi.nlm.nih.gov/pubmed/11313899 10.1038/sj.onc.1204184 11313899

[28] Cobb MH, Goldsmith EJ. How MAP kinases are regulated. J Biol Chem. 1995;270:14843–6. 7797459 10.1074/jbc.270.25.14843

[29] Kim J-Y, Kim Y-J, Lee S, Park J-H. The critical role of ERK in death resistance and invasiveness of hypoxia-selected glioblastoma cells. BMC Cancer. 2009;9:27 doi: 10.1186/1471-2407-9-27 19161638 10.1186/1471-2407-9-27 PMC2645423

[30] Rasola A, Sciacovelli M, Chiara F, Pantic B, Brusilow WS, Bernardi P. Activation of mitochondrial ERK protects cancer cells from death through inhibition of the permeability transition. Proc Natl Acad Sci U S A [Internet]. 2010;107(2):726–31. Disponible en: http://www.pubmedcentral.nih.gov/articlerender.fcgi?artid=2818893&tool=pmcentrez&rendertype=abstract 10.1073/pnas.0912742107 PMC2818893 20080742

[31] Baines CP, Zhang J, Wang GW, Zheng YT, Xiu JX, Cardwell EM, et al Mitochondrial PKCε and MAPK form signaling modules in the murine heart: Enhanced mitochondrial PKCε-MAPK interactions and differential MAPK activation in PKCε-induced cardioprotection. Circ Res. 2002;90(4):390–7. 11884367 10.1161/01.res.0000012702.90501.8d

[32] Majumder PK, Pandey P, Sun X, Cheng K, Datta R, Saxena S, et al Mitochondrial translocation of protein kinase C delta in phorbol ester-induced cytochrome c release and apoptosis. J Biol Chem. 2000;275(29):21793–6. doi: 10.1074/jbc.C000048200 10818086 10.1074/jbc.C000048200

[33] Robbins DJ, Zhen E, Owaki H, Vanderbilt CA, Ebert D, Geppert TD, et al Regulation and properties of extracellular signal-regulated protein kinases 1 and 2 in vitro. J Biol Chem. 1993;268(7):5097–106. 8444886

[34] Satoh Y, Kobayashi Y, Takeuchi A, Pagès G, Pouysségur J, Kazama T. Deletion of ERK1 and ERK2 in the CNS causes cortical abnormalities and neonatal lethality: Erk1 deficiency enhances the impairment of neurogenesis in Erk2-deficient mice. J Neurosci [Internet]. 2011;31(3):1149–55. Disponible en: http://www.ncbi.nlm.nih.gov/pubmed/21248139 10.1523/JNEUROSCI.2243-10.2011 PMC6632941 21248139

[35] Arciuch VGA, Alippe Y, Carreras MC, Poderoso JJ. Mitochondrial kinases in cell signaling: Facts and perspectives. Vol. 61, Advanced Drug Delivery Reviews. 2009 p. 1234–49. 10.1016/j.addr.2009.04.025 19733603

[36] Monick MM, Powers LS, Barrett CW, Hinde S, Ashare A, Groskreutz DJ, et al Constitutive ERK MAPK activity regulates macrophage ATP production and mitochondrial integrity. J Immunol [Internet]. 2008;180(11):7485–96. Disponible en: http://www.pubmedcentral.nih.gov/articlerender.fcgi?artid=2410094&tool=pmcentrez&rendertype=abstract 10.4049/jimmunol.180.11.7485 PMC2410094 18490749

